# Study on Isomeric Impurities in Cefotiam Hydrochloride

**DOI:** 10.3389/fchem.2020.619307

**Published:** 2021-01-15

**Authors:** Ye Tian, Xiao-Meng Chong, Ying Liu, Ying Han, Chang-Qin Hu, Shang-chen Yao, Ming-Zhe Xu

**Affiliations:** National Institutes for Food and Drug Control, Beijing, China

**Keywords:** cefotiam hydrochloride, column switching, HPLC-MS, isomer, nuclear magnetic resonance

## Abstract

In this study, two isomeric impurities were identified in cefotiam hydrochloride injection preparation and were characterized. Column-switching HPLC-MS and NMR techniques were used to identify the impurity 1 as the Δ3(4) isomers of cefotiam. Using software-based calculations, it was predicted that neither of the isomeric impurities was embryotoxic. This study provides a reference for the production, storage, and quality control of cefotiam and related cephalosporin antibiotics.

## Introduction

**Figure 1:**
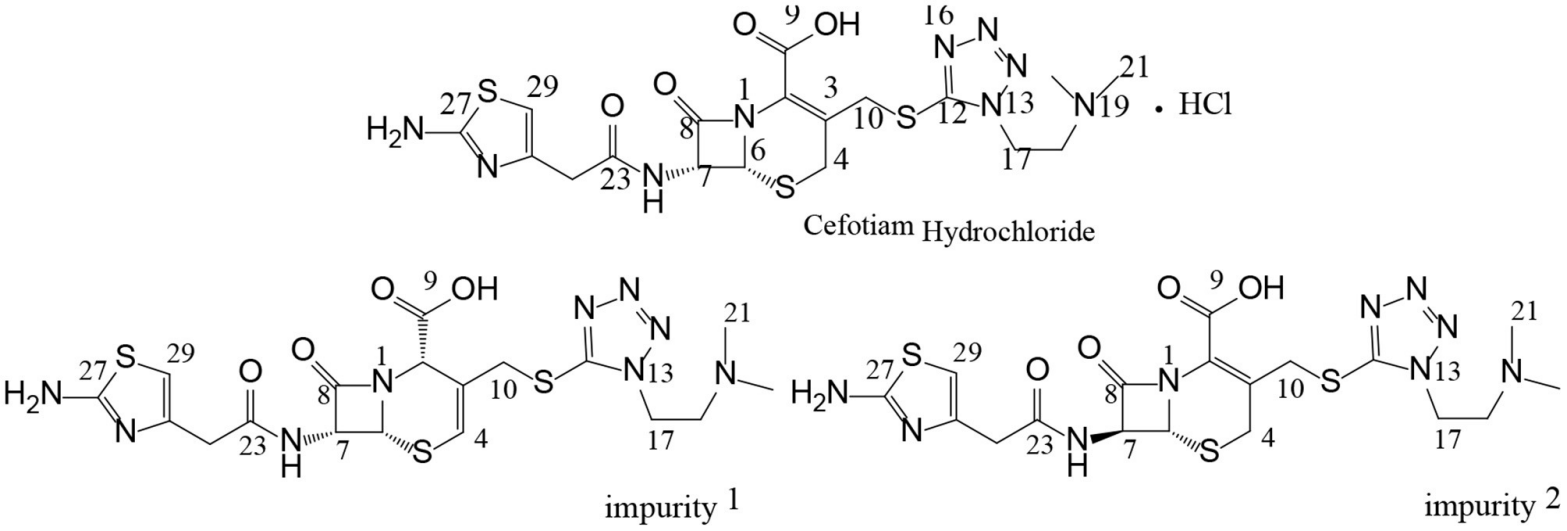
Chemical structures of cefotiam, impurity 1, and impurity 2.

Cefotiam hydrochloride (Figure 1) is included in both the USP 40 and the Japanese Pharmacopeia 17. It is a second-generation cephalosporin antibiotic which is effective against both Gram-positive and Gram-negative bacteria (Japanese Pharmacopeia The Society of Japanese Pharmacopeia, 2016; United States Pharmacopeial Convention, 2017). There have been some studies on impurities in cefotiam preparations for injection (Tang et al., 2013; Yang, 2013; Tian et al., 2018). These studies on the impurity profiles form the basis for drug quality control and research. The International Council for Harmonization of Technical Requirements for Pharmaceuticals for Human Use (ICH) requires that impurities present in concentrations >0.1% be structurally identified, thus providing a guarantee for product safety, efficacy, and quality control. Reversed-phase liquid chromatography (RP–LC) is still the primary method for analyte separation toward impurity profiling of drugs. However, an increasing number of examples show the usefulness of mass spectrometry (MS) as additional tools for the detection and quantitation of difficult to solve impurity challenges. However, cephalosporin antibiotics contain multiple chiral centers, and the C-3 and C-7 side chains often contain heteroatoms (Figure 1). The stereoisomeric and constitutional isomeric impurities that may be formed during the production and storage processes can reduce drug efficacy or enhance toxicity, and it is relatively difficult to determine the structures of isomeric impurities by the aforementioned techniques (Okamoto et al., 1996a,b; Jiang et al., 2010; Liu et al., 2010; Tian et al., 2015a,b).

Previously, our group studied the structures, sources, and toxicities of two constitutional isomeric impurities in cefotiam hydrochloride (Tian et al., 2018). In further studies, two other isomeric impurities were discovered in long-term-stability samples produced by Shanghai Xinya Pharmaceutical Co., Ltd. In this paper, we report the comprehensive analysis and structural study of these two isomeric impurities using column-switching LC/MS and NMR, as well as software-based toxicity prediction. Thus, this study contributes to the understanding and design of quality control processes for cefotiam and related cephalosporin antibiotics.

## Materials and Methods

### Forced Degradation Studies

For the forced degradation studies, the samples were subjected to stress conditions according to ICH guidelines. For acidic and alkaline degradation, 20 mg of cefotiam hydrochloride was dissolved in 1.0 mL of 0.1 M HCl or NaOH and maintained at room temperature for 3 h. Before further dilution, these samples were neutralized. For degradation by oxidation, 20 mg of cefotiam hydrochloride was dissolved in 1.0 mL of 10% H_2_O_2_ and stored at room temperature for 20 min. To investigate the degradation of isolated impurities at high temperatures, the samples were heated at 60°C for 5 days or at 80°C for 12 h in an oven, removed, and allowed to cool to room temperature before further analysis. Finally, the samples were diluted with mobile phase A described in 2.3.1 to a final concentration of 2 mg·mL^−1^ cefotiam hydrochloride.

### Instruments and Chemicals

The following instruments were used in our study: a Shimadzu LC-20AT pump with an SPD-M20A photodiode array detector and LabSolutions workstation (Shimadzu Corporation, Kyoto, Japan), a Q-Trap 3200 (AB Sciex, Foster City, CA, USA), and a Varian INOVA 600 MHz NMR spectrometer (Varian, Palo Alto, CA, USA). HPLC-grade methanol and acetonitrile as well as disodium hydrogen phosphate, potassium dihydrogen phosphate, and potassium hydroxide were obtained from Sigma-Aldrich (St. Louis, MO, USA). Formic acid was purchased from Fluka (Buchs, Switzerland). Water was deionized with a purification system form Milli-Q reference (Billerica, MA, USA).

### Chromatographic and Mass Spectrometric Conditions

#### The HPLC System

The first HPLC separation was carried out at a constant column temperature of 40°C, using a detection wavelength of 254 nm with an Kromasil reversed-phase C18 column (4.6 × 250 mm, 5 μm). The flow rate of the mobile phase and the injection volume were 1.0 mL/min and 20 μL, respectively. The gradient elution program used is described in Table 1 (Tian et al., 2018). Phase A: PBS solution (7.098 g of anhydrous sodium hydrogen phosphate was dissolved in water, and diluted to 1,000 mL, to 800 mL of this solution, 6.80 g of potassium dihydrogen phosphate was added, and diluted with water to 1,000 mL, and the pH was adjusted to 7.2); phase B: acetonitrile.

**Table 1:** Gradient elution program for separatory HPLC.

**Time (min)**	**Mobile phase A (%, v/v)**	**Mobile phase B (%, v/v)**
0	97	3
20	90	10
50	75	25
51	97	3
55	97	3

#### Column-Switching LC/MS Method

**Figure 2:**
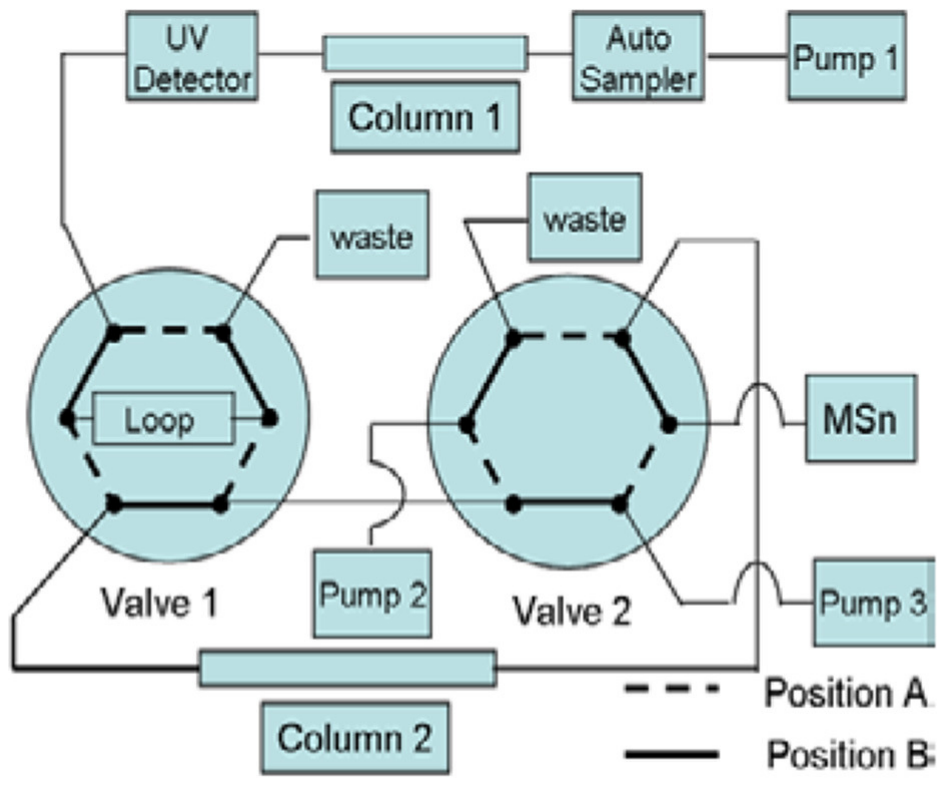
Schematic diagram of column switching.

First-dimensional Chromatographic System: This experiment used the same method as given in Part 2.3.1, the RPHPLC method. It was used for the separation of impurities. Second-dimensional Chromatographic System: The analysis was carried out on a CAPCELL PAK C18 (150 × 4.6 mm, 5 μm) column. The pump mode was the gradient, and the program described in Table 2. The column oven temperature was set at 30°C. The flow rate was kept at 0.5 ml·min^−1^. The switching valves were 6-way valves, A and B. The quantitative ring volume for switching was 500 μl. This system was used for the desalination of impurities during the mass spectrometry analysis (Figure 2). Mobile phase C: 0.3% formic acid aqueous solution; phase D: 90:10 methanol-0.3% formic acid aqueous solution.

**Table 2:** Gradient elution program for LC/MS.

**Time (min)**	**Mobile phase C (%, v/v)**	**Mobile phase D (%, v/v)**
0.0	100	0
5.5	100	0
5.6	100	0
25	0	100
30	0	100

Mass spectrometry was performed in positive ion mode using an electrospray ionization (ESI) ion source. The conditions used for MS analysis were as follows: electrospray voltage: 4,500 V; curtain Gas flow: 20; ion source temperature: 500°C, ion source gas 1: 60; ion source gas 2: 65; collision gas: high; declustering potential: 71.0; collision energy: 10.00; collision exit potential: 3.00.

### Preparative Liquid Chromatography

A Grace Prevail C18 column (5 μm, 10 mm × 250 mm i.d.) was used for separation by the method described in Table 1 at a flow rate of 2.0 mL/min. The column eluent was monitored at a wavelength of 254 nm. The injection volume was 50 μL.

### NMR Measurements

1D- and 2D-NMR measurements were performed on a Varian INOVA 600 MHz instrument at 25°C in DMSO-*d*_6_. The ^1^H and ^13^C chemical shifts values are reported on the δ scale in ppm relative to TMS (δ = 0.00) and DMSO-*d*_6_ (δ = 39.5 ppm), respectively, as the internal standards.

### Molecular Docking

Docking was studied with Discovery Studio 4.0 software package (Accelrys Software Inc., CA, USA). By the homology modeling server SWISS-MODEL (https://swissmodel.expasy.org/), the three-dimensional (3D) crystallographic structure of the hyaluronan synthase 1 (HAS1) protein was generated. Before docking, hydrogen atoms were added to the unoccupied valence of the heavy atoms of the protein. The HAS1 protein was defined as a receptor using DS 4.0. For ligand preparation, the structures of the experimental compounds were downloaded from the PubChem Compound Database (https://www.ncbi.nlm.nih.gov/pccompound/). From the receptor–ligand interaction section of DS 4.0, the CDOCKER protocol was selected and used in the docking studies. Docking was performed using a simulated annealing method to minimize the CDOCKER energy and to obtain the optimum position.

## Results and Discussion

### HPLC Analysis of Impurities and Degradation Tests

**Figure 3:**
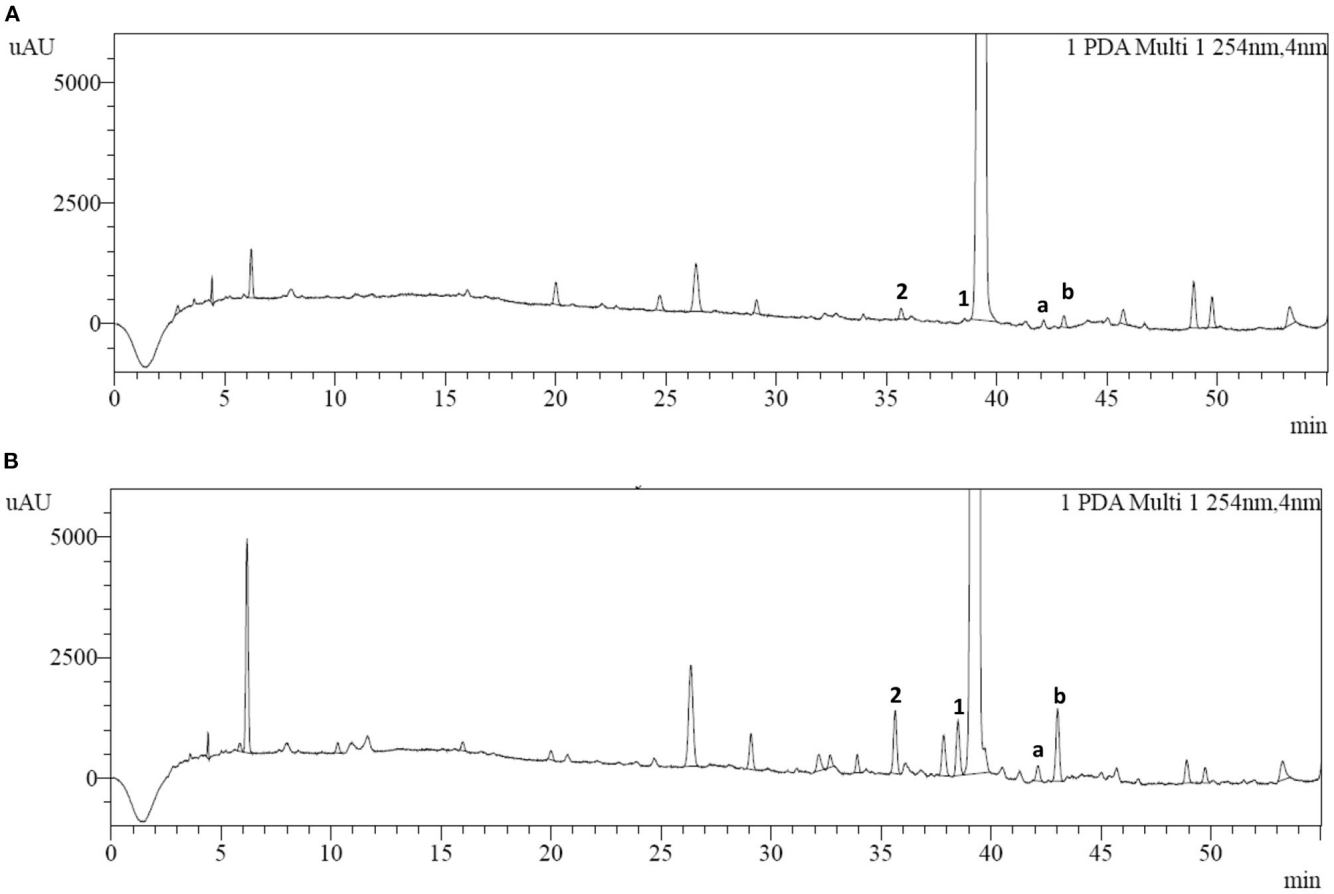
HPLC chromatograms of **(A)** cefotiam hydrochloride and **(B)** the long-term-stability sample of cefotiam hydrochloride preparation.

HPLC analysis of cefotiam hydrochloride preparations for injection was conducted according to the method described in 2.3 (Figure 3). Two impurities (impurities 1 and 2) were detected and their quantities in the long-term-stability samples were increased to 0.15% and 0.17%, respectively, from 0.02 to 0.06%, respectively, in the fresh samples, as calculated by the normalized method. The samples were then subjected to various degradation experiments. After being maintained at 60°C for 5 days or at 80°C for 12 h, the content of impurity 1 increased significantly relative to the content in the long-term-stability sample, indicating that impurity 1 is susceptible to degradation under high-temperature conditions. However, the content of impurity 2 did not change significantly under high-temperature, acidic, or alkaline conditions, or other harsh experimental conditions.

### Structural Analysis of Impurities 1 and 2

Liquid chromatography mass spectrometry (LC–MS) is often used for detecting the impurities in drugs, while the reported methods often used non-volatile salt, which is not suitable for the MS system. By the two-dimensional liquid chromatography-mass spectrometry (2D-LC–MS/MS) technology, pharmaceuticals can be analyzed in the first dimensional LC system using mass incompatible mobile phases, and then transferred to the second LC system to capture and desalt the compounds which expands the application range of mass spectrometry. This technology has been applied to this research.

**Figure 4:**
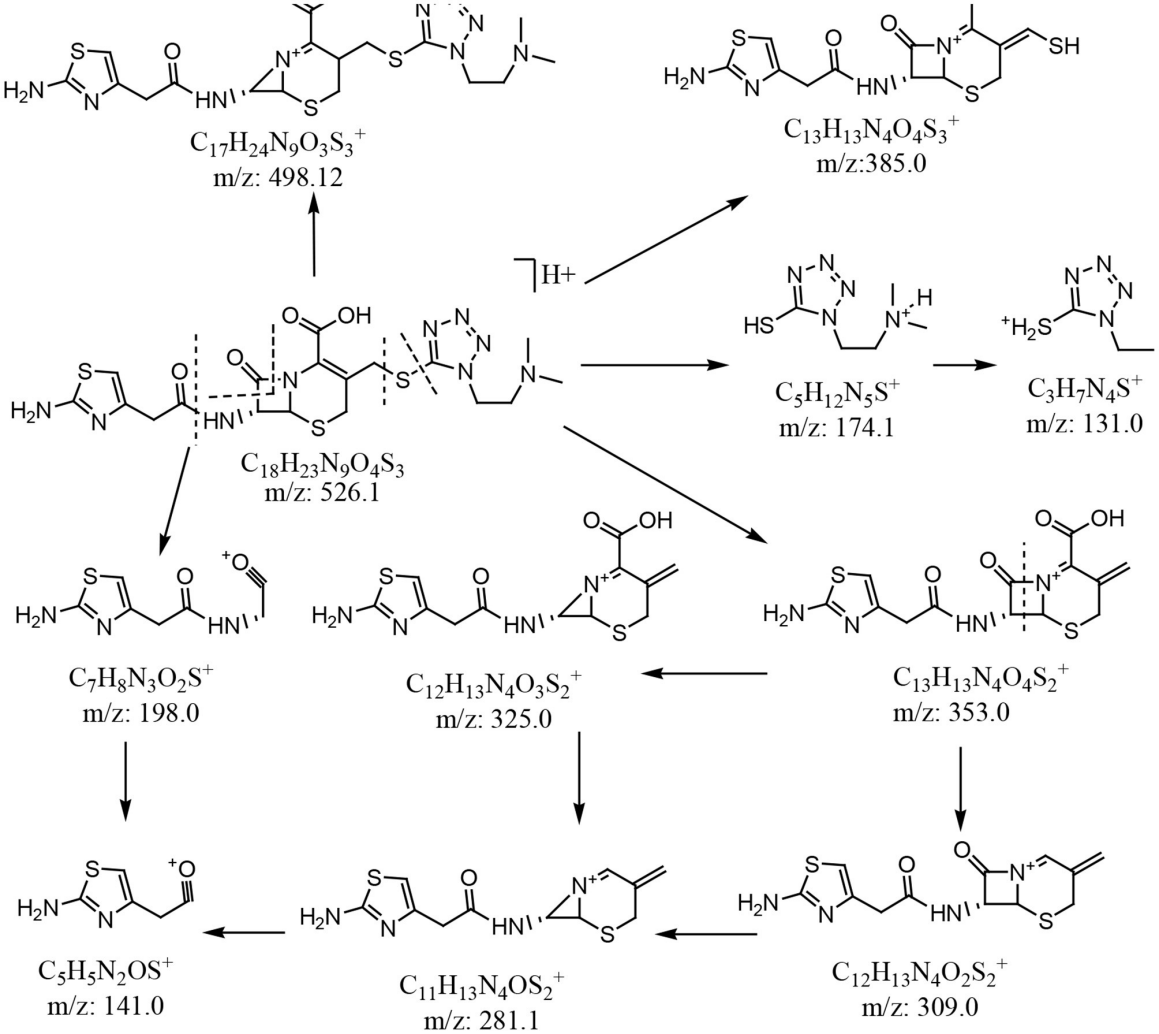
Plausible MS fragmentation pattern for cefotiam.

In the enhanced mass spectrum (positive ion mode) of impurity 1, peaks were identified at *m/z* 526.3 ([M+H]^+^) and *m/z* 548.2 ([M+Na]^+^). Based on this data, it was determined that the molecular weight of impurity 1 was 525, which is the same as that of cefotiam. The enhanced product ion spectrum obtained in positive ion mode for the *m*/*z* 526.3 peak displayed the characteristic fragments of cefotiam (*m/z* 198.1, 385.0, and 498.1; Figure 4) (Tian et al., 2018). Thus, it was speculated that impurity 1 was an isomer of cefotiam.

The structure of impurity 1 could not be determined using only the MS data. By exploring the behavior of impurity 1 under different conditions, it was found that its content increased significantly when cefotiam was kept at high temperature (120°C) for 5 h, and it could be purified by preparative HPLC, allowing the structures to be determined by analysis of NMR spectra.

The ^1^H NMR data of cefotiam was assigned to accurately resolve the structure of impurity 1. ^1^H NMR data (600 MHz, DMSO-*d*_6_): δ (ppm) 6.25 (1H, s, H-29), 4.95 (1H, d, *J* = 4.8 Hz, H-6), 5.54 (1H, dd, *J* = 4.8 Hz, 8.4, H-7), 3.59 (1H, d, *J* = 17.4 Hz, H-4a), 3.32 (1H, d, *J* = 17.4 Hz, H-4b), 4.37 (1H, d, *J* = 12.6 Hz, H-10a), 4.26 (1H, d, *J* = 12.6 Hz, H-10a), 2.16 (6H, s, H_3_-20, 21). The ^13^C DEPT spectrum of cefotiam showed 18 signals, corresponding to 8 fully substituted, 3 methine, 5 methylene, and 2 methyl carbons. The assignment of ^1^H and ^13^C NMR spectra of cefotiam was assisted by HSQC, HMBC, NOESY, and other techniques, as shown in Table 3.

**Table 3:** NMR data for cefotiam and impurity 1 in DMSO-*d*_6_^a^.

**Peak No**.	**Cefotiam**	**Impurity 1**	**Cefotiam**	**Impurity 1**
2		4.54, 1H, s	113.4 (CH)	53.4 (CH)
3		6.24, 1H, s,	133.4 (C)	123.4 (C)
4	3.59, 1H, d, *J* = 17.4		26.2 (CH_2_)	115.3 (CH)
	3.32, 1H, d, *J* = 17.4			
6	4.95, 1H, d, *J* = 4.8	5.22, 1H, d, *J* = 4.8	57.1 (CH)	53.0 (CH)
7	5.54, 1H, dd, *J* = 4.8, 8.4	5.23, 1H, dd, *J* = 4.8, 7.8	58.5 (CH)	59.7 (CH)
8			163.4 (C)	162.4 (C)
9			166.8 (C)	166.8 (C)
10	4.37, 1H, d, *J* = 12.6	4.15, 1H, d, *J* = 13.2	39.0 (CH_2_)	39.0 (CH_2_)
	4.26, 1H, d, *J* = 12.6	4.54, 1H, d, *J* = 13.2		
12			154.8 (C)	153.6 (C)
17	4.37, 2H, t, *J* = 6.0	4.38, 2H, m	45.1 (CH_2_)	45.1 (CH_2_)
18	2.69, 2H, t, *J* = 6.0	2.67, 2H, t, *J* = 6.0	56.9 (CH_2_)	57.2 (CH_2_)
20	2.16, 3H, s	2.15, 3H, s	45.0 (CH_3_)	45.0 (CH_3_)
21	2.16, 3H, s	2.15, 3H, s	45.0 (CH_3_)	45.0 (CH_3_)
22	8.76, 1H, d, *J* = 8.4	8.80, 1H, d, *J* = 7.8		
23			169.9 (C)	169.8 (C)
24	3.37, 2H, s	3.34, 2H, s	37.1 (CH_2_)	37.9 (CH_2_)
25			145.6 (C)	145.6 (C)
27			168.3 (C)	168.1 (C)
29	6.25, 1H, s	6.22, 1H, s	102.5 (CH)	102.5 (CH)
30	6.89, 2H, s	6.87, 2H, s		

a *Data (δ) were measured at 600 MHz. Coupling constants (J) in Hz are given in parentheses. The assignments were based on distortionless enhancement by polarization transfer (DEPT), ^1^H-^1^H COSY, HSQC, and HMBC experiments*.

The ^1^H NMR spectrum of impurity 1 was then analyzed. ^1^H NMR data (600 MHz, DMSO-d_6_): δ (ppm) 6.22 (1H, s, H-29), 5.22 (1H, d, *J* = 4.8 Hz, H-6), 5.23 (1H, dd, J1 = 4.8 Hz, J2 = 7.8 Hz, H-7), 4.15 (1H, d, *J* = 13.2 Hz, H-10a), 4.54 (1H, d, *J* = 13.2 Hz, H-10b). The signals at 5.22 and 5.23 ppm are attributed to adjacent protons, characteristic of the 4-membered lactam ring of cephalosporin, suggesting that the 4-membered lactam ring is maintained in impurity 1 with no change in the configuration of C-7 (Okamoto et al., 1996a). The main difference between the ^1^H NMR spectra of impurity 1 and cefotiam is that the two methine signals (6.24 and 4.54 ppm) in impurity 1 replace the single methylene signal in cefotiam. This was also confirmed by the ^13^C NMR and DEPT spectra. Peaks at 53.4 ppm (CH, C-2), 123.4 ppm (>C <, C-3), and 115.3 ppm (CH, C-4) replaced those corresponding to one methine and two fully substituted carbon atoms of cefotiam, the peaks corresponding to C-6 and C-8 were shifted upfield by 4.1 and 1.0 ppm, respectively, and that corresponding to C-7 was shifted downfield by 1.2 ppm. Further, the characteristic UV absorption peak of cefotiam at 260 nm was shifted to 248 nm, suggesting that the conjugation system of the compound had changed. It was thus speculated that impurity 1 was a product of the migration of a double bond in cefotiam.

The structure of impurity 1 was further confirmed by HSQC and HMBC experiments. In the HMBC spectrum, H-4 was correlated with C-2, C-6, and C-10, H-10 was correlated to C-2, C-3, C-4, and C-12, and H-17 was correlated to C-12 and C-18. It was thus confirmed that impurity 1 is the Δ3(4) isomer of cefotiam (Figure 1 and Table 3). Based on previously reported data, it was determined that C-2 of impurity 1 was in the *S* configuration (Van Heyningen and Ahern, 1968).

In the enhanced mass spectrum (positive ion mode) of impurity 2, peaks were identified at *m/z* 526.3 ([M+H]^+^) and 548.3 ([M+Na]^+^). It was thus determined that the molecular weight of the impurity was 525, which is the same as that of cefotiam, suggesting that impurity 2 is an isomer of cefotiam. The enhanced product ion spectrum obtained in positive ion mode for the *m/z* 526.3 peak exhibited the characteristic fragments of cefotiam (*m/z* 385, 353, 309, 281, 198, 174, and 131; Figure 4) (Tian et al., 2018). Since the UV absorbance spectrum still exhibited a peak at 260 nm, it was suggested that impurity 2 retained the core structure of cefotiam, i.e., the double bond in the dihydrothiazide ring was not displaced to form the Δ3(4) isomer. Because of the structural characteristics of cephalosporin compounds, C-7 is the only chiral center (among C-2, C-6, and C-7) that could be isomerized during production and storage; this would result in the 7-*S* isomer (Tian et al., 2014). Therefore, impurity 2 was speculated to be the 7-*S* isomer of cefotiam (Figure 1).

### Embryotoxicity Predictions for Impurities 1 and 2

Based on our previous studies, it is known that 5-mercapto-1-methyltetrazole (MTT) and 5-mercapto-1-dimethylaminoethyltetrazole (DMMT) bind to the HAS1 protein in zebrafish (*Danio rerio*) embryos, causing embryotoxicity. Further, changes to the C-7 side chain of cephalosporin antibiotics may affect the binding of MTT and DMMT to HAS1. Therefore, the HAS1 protein was selected for docking experiments to investigate the embryotoxicity of the cefotiam impurities (Han et al., 2018).

**Figure 5:**
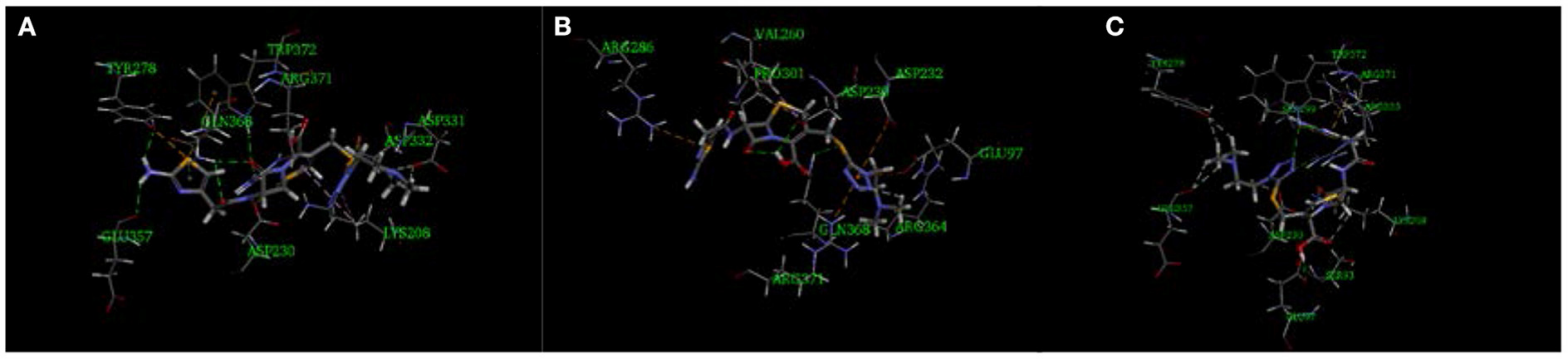
Three-dimensional ligand docking model, showing the interactions of **(A)** cefotiam, **(B)** impurity 1, and **(C)** impurity 2 with residues in the active site of the HAS1 protein. The arrows indicate hydrogen bonds.

The imidogen and carboxyl moieties of cefotiam formed five hydrogen bonds with Asp 230, Gln 368, Arg 371, and Trp 372 residues, and the amino group of the C-7 side chain formed two hydrogen bonds with Glu 357 and Typ 278 (Figure 5A) (Tian et al., 2018). The S-11 atom of the C-3 side chain of impurity 1 and the carboxyl on the mother nucleus formed hydrogen bonds with Gln 368 and Asp 230 (Figure 5B). In impurity 2, the carboxyl group and the nitrogen atoms in the C-3 and C-7 side chains formed three hydrogen bonds with Sep 299, Glu 97, and Ser 93 residues (Figure 5C). CDOCKER predictions (Table 4) did not indicate any significant embryotoxicity arising from impurities 1 and 2.

**Table 4:** Docking results.

**Protein**	**Compound**	**-CDOCKER Energy**	**-CDOCKER Interaction Energy**
HAS1	Cefotiam	−20.4585	56.1571
HAS1	impurity 1	−4.1616	57.0408
HAS1	impurity 2	−5.2601	55.9512

## Conclusion

In this study, column-switching LC/MS and NMR were used to determine the presence and identities of two isomeric impurities in long-term-stability samples of cefotiam for injection. Between these two isomers, only impurity 1 [the Δ3(4) isomer of cefotiam] was shown to degrade under high-temperature conditions. Software calculations predicted that neither impurity would be significantly embryotoxic. This study provides a basis for structural studies of impurities and quality control considerations in cefotiam and related cephalosporin antibiotics.

## Data Availability Statement

The original contributions presented in the study are included in the article/Supplementary Materials, further inquiries can be directed to the corresponding author/s.

## Author Contributions

YT: specific experimental work and experimental design. X-MC and YH: specific experimental work. S-cY and M-ZX: experimental design. YL: Preparative liquid chromatography NMR measurements. C-QH: Design of experiments. All authors contributed to the article and approved the submitted version.

## Conflict of Interest

The authors declare that the research was conducted in the absence of any commercial or financial relationships that could be construed as a potential conflict of interest.
