# Supplementary material for: Study on Isomeric Impurities in Cefotiam Hydrochloride
Source: Front Chem. 2021 Jan 15;8:619307. doi: 10.3389/fchem.2020.619307 (PMC7873999; doi:10.3389/fchem.2020.619307)
Supplement: Supplementary file 1 [file Table_1.DOC]

**Study on Isomeric Impurities in Cefotiam Hydrochloride**

Tian Ye, Xiao-Meng Chong, Shang-chen Yao* Ming-Zhe Xu*

National Institutes for Food and Drug Control, Beijing 102629, China

**Supplementary Content**

**List of Contents**

| No. | Content | Page |
| --- | --- | --- |
| 1 | **Figure S1.** The ^1^H NMR Spectrum of impurity 1 in DMSO-*d_6_* (600 MHz). | S3 |
| 2 | Figure S2. The ^13^C NMR Spectrum of impurity 1 in DMSO-*d_6_* (150 MHz). | S4 |
| 3 | Figure S3. The DEPT Spectrum of impurity 1 in DMSO-*d_6_* (150 MHz). | S5 |
| 4 | Figure S4. The ^1^H-^1^H gCOSY Spectrum of impurity 1 in DMSO-*d_6_* (600 MHz). | S6 |
| 8 | Figure S5. The gHSQC Spectrum of impurity 1 in DMSO-*d_6_* (600 MHz). | S7 |
| 9 | Figure S6. The gHMBC Spectrum of impurity 1 in DMSO-*d_6_* (600 MHz). | S8 |


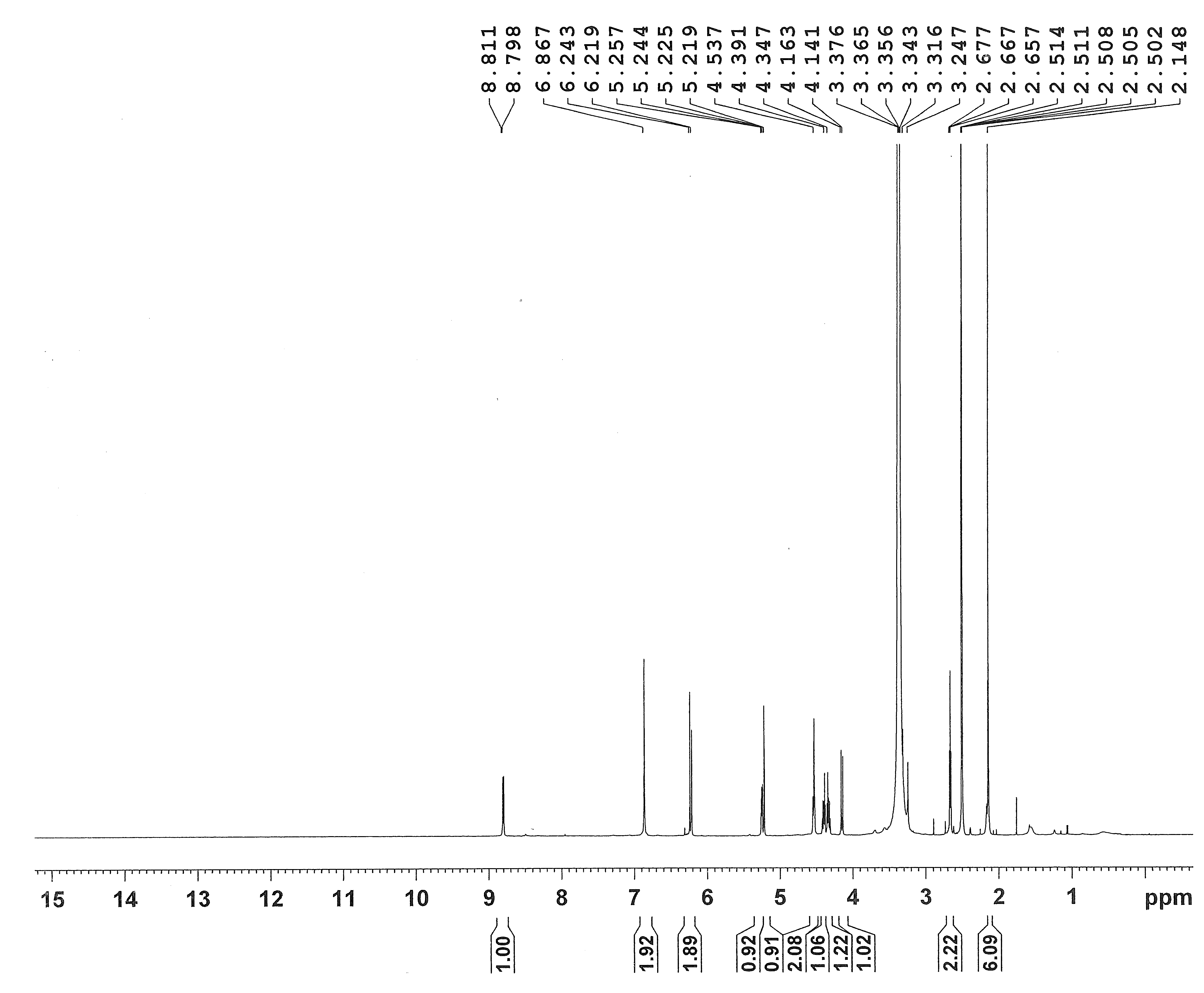


Figure S1. The ^1^H NMR Spectrum of impurity 1 in DMSO-*d_6_* (600 MHz).


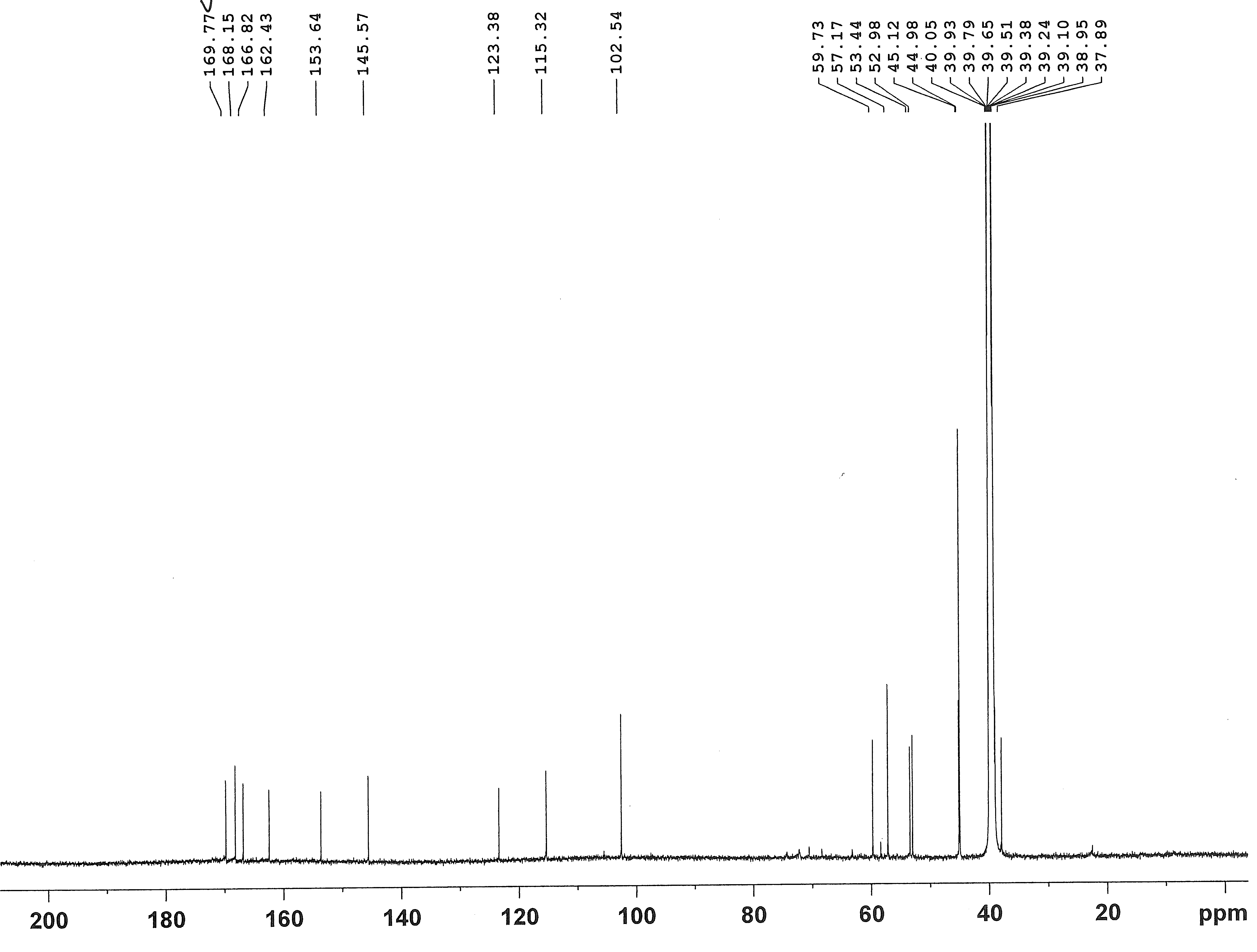


Figure S2. The ^13^C NMR Spectrum of impurity 1 in DMSO-*d_6_* (150 MHz).


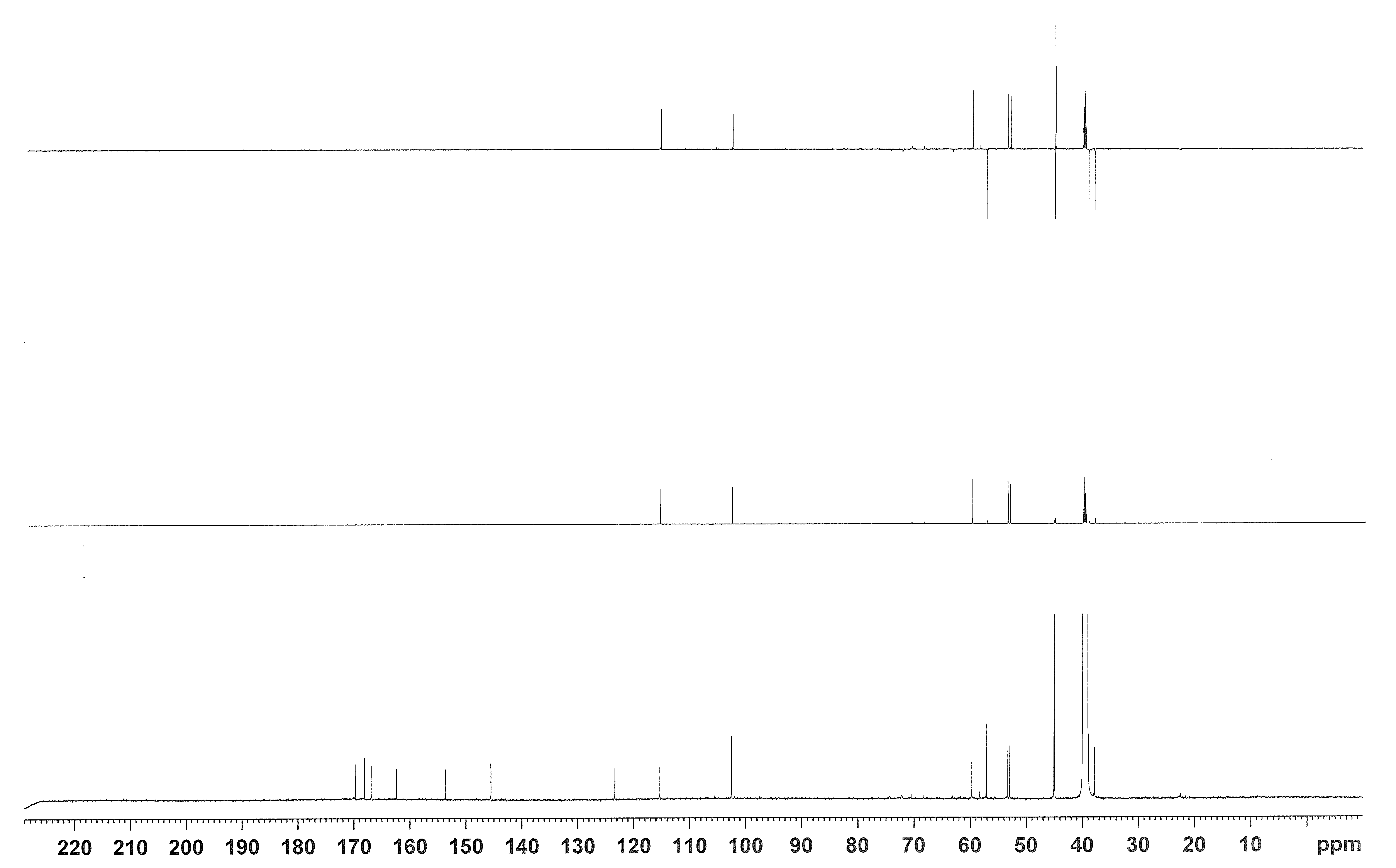


Figure S3. The DEPT Spectrum of impurity 1 in DMSO-*d_6_* (150 MHz)


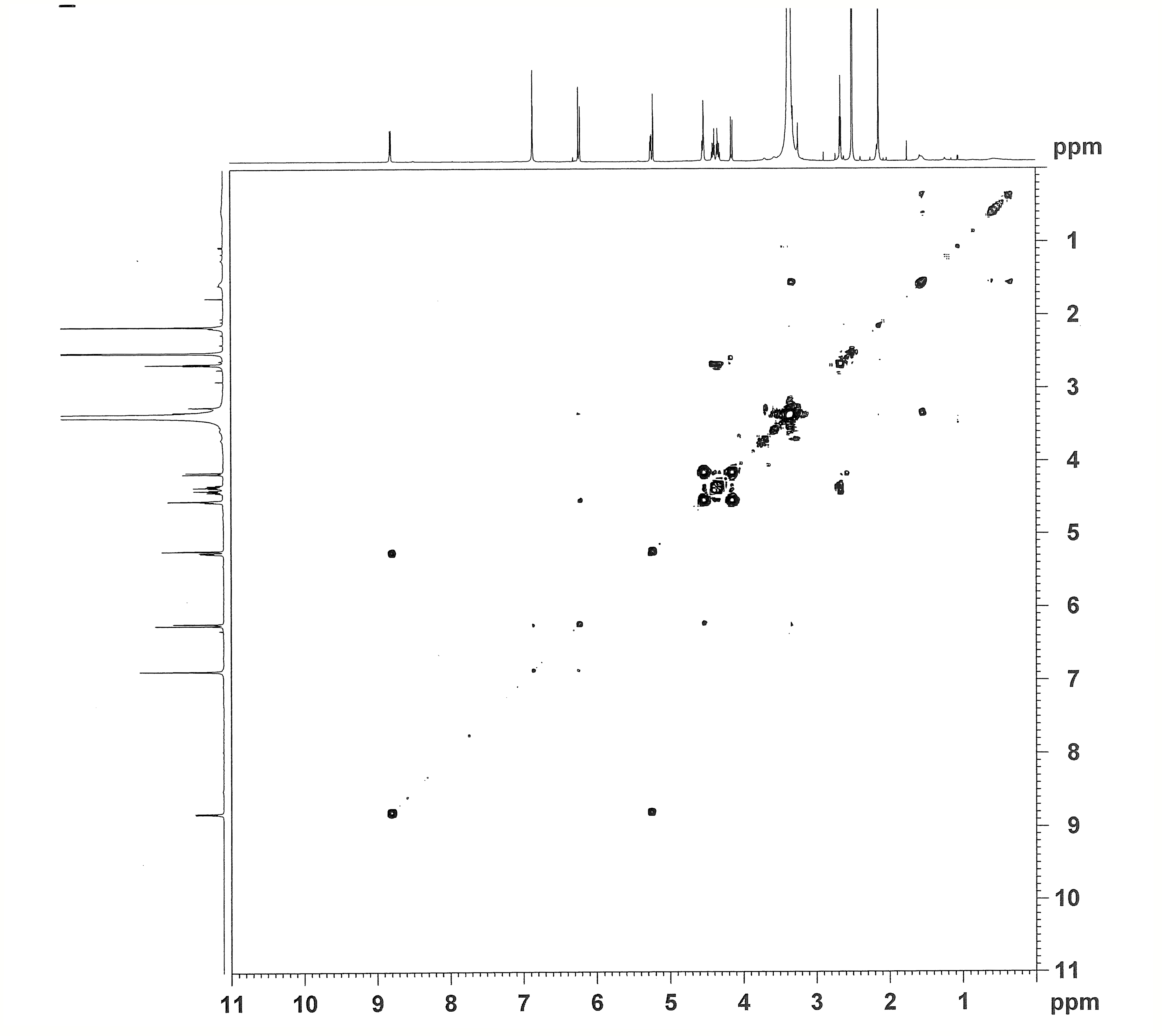


Figure S4. The ^1^H-^1^H gCOSY Spectrum of impurity 1 in DMSO-*d_6_* (600 MHz).


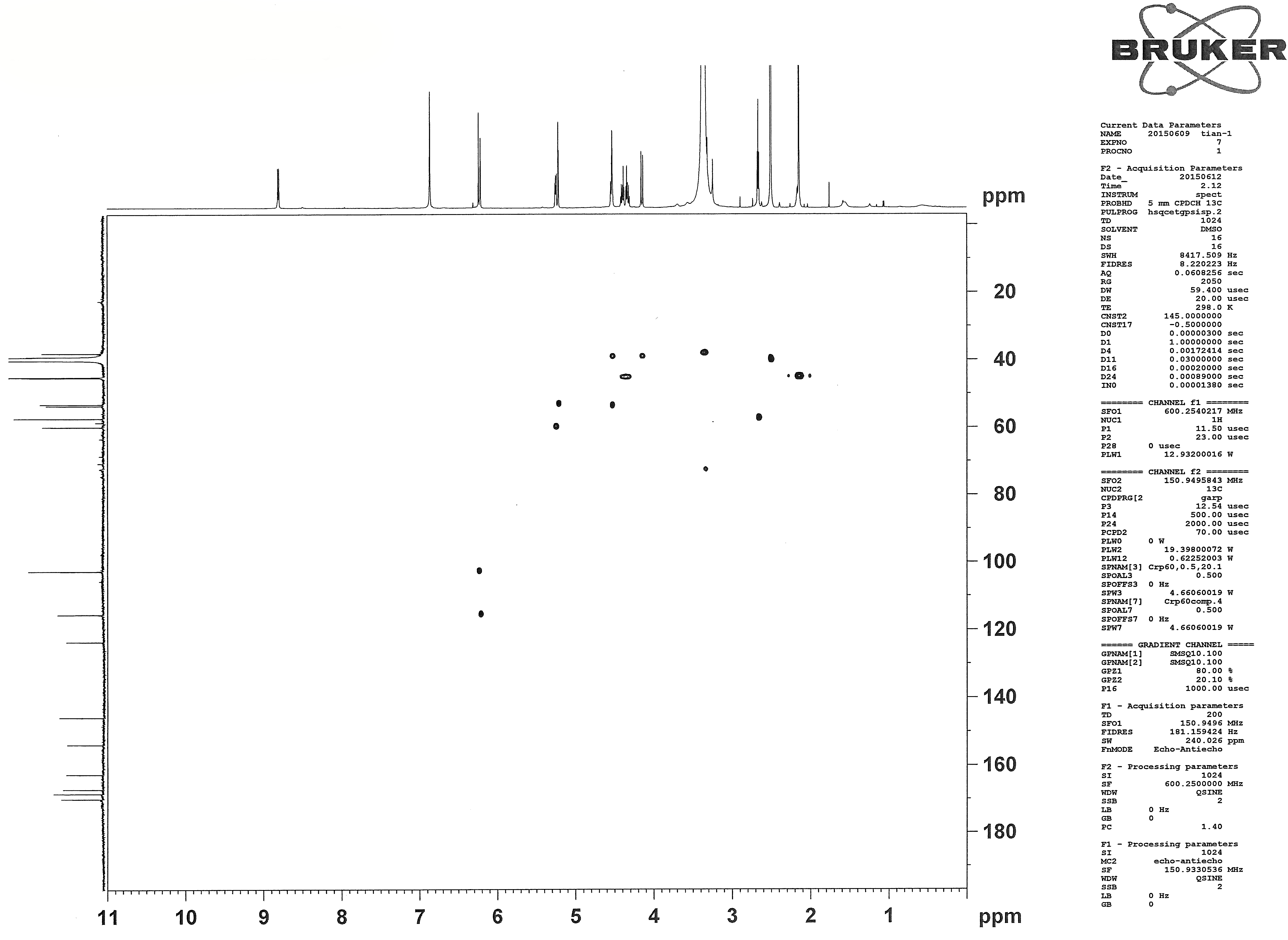


Figure S5. The gHSQC Spectrum of impurity 1 in DMSO-*d_6_* (600 MHz).


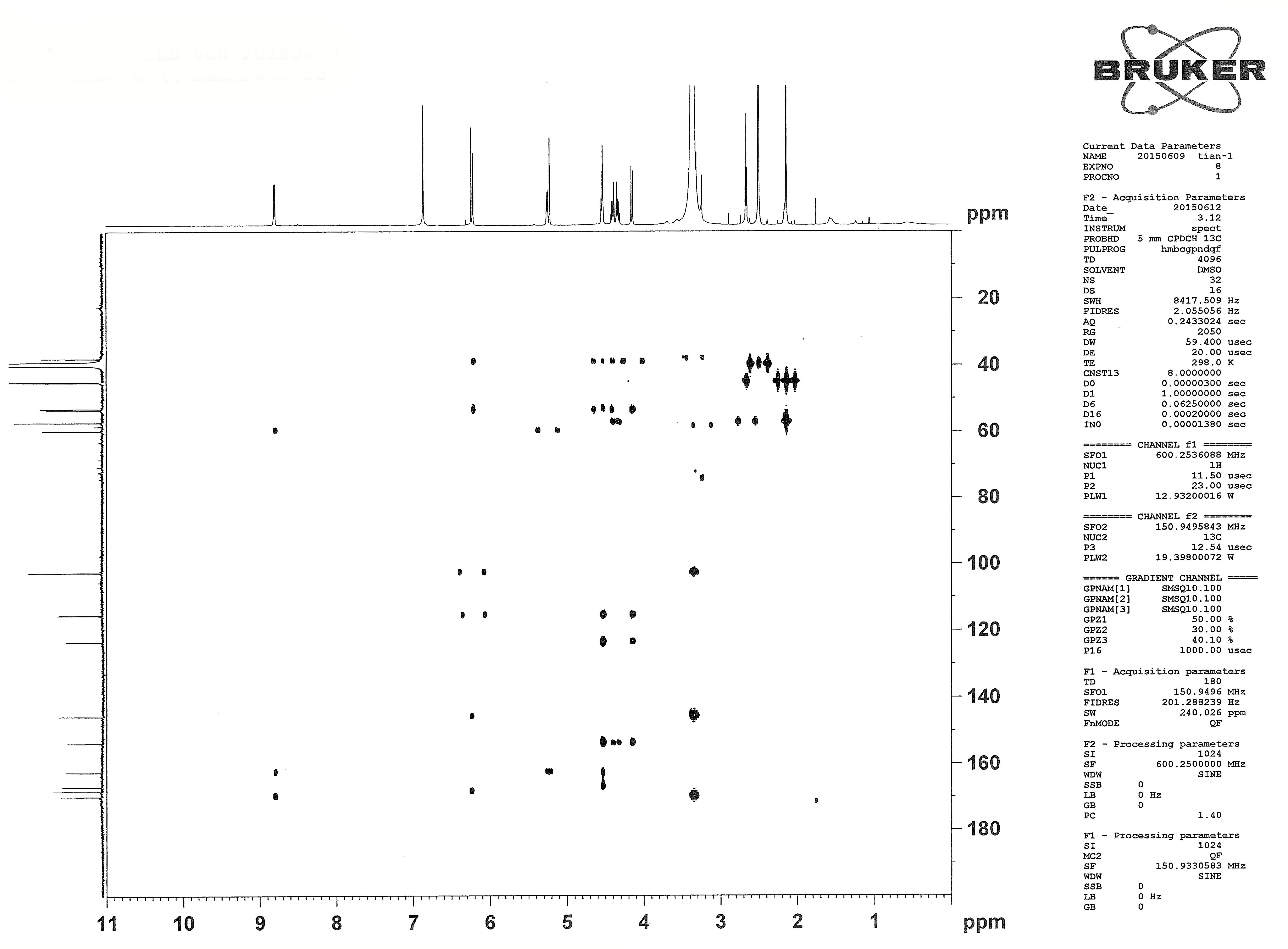


Figure S6. The gHMBC Spectrum of impurity 1 in DMSO-*d_6_* (600 MHz).
